# What school nurses strive to achieve for themselves in order to remain in practice: A qualitative study

**DOI:** 10.1002/nop2.1333

**Published:** 2022-08-21

**Authors:** Linda Horne Mæland, Bjørg Frøysland Oftedal, Margareth Kristoffersen

**Affiliations:** ^1^ Faculty of Health Science University of Stavanger Stavanger Norway

**Keywords:** goods of higher value, nurse, phenomenological hermeneutic method, remaining in nursing, school nurse, striving to achieve

## Abstract

**Aim:**

This study aims to describe and interpret what it is school nurses strive to achieve for themselves in order to remain in practice.

**Design:**

A qualitative study with a hermeneutic approach.

**Method:**

The data were collected by means of in‐depth interviews with 15 Norwegian school nurses on two separate occasions and analysed using a phenomenological hermeneutic method.

**Results:**

The analysis resulted in the following themes: (1) trusting your own professional ability, (2) aspiring to appreciation and (3) accomplishing self‐care. These themes were reflected in the school nurses' choices and actions and were regarded as an expression of what was of value to them as a nurse. Thus, the nurses' realizing what they strived to achieve for themselves can comprehensively be understood as a good of higher value for their remaining in nursing practice.

## 1 INTRODUCTION

There seems to be a link between the goods nurses strive to achieve on their own behalf and their remaining in practice (Kristoffersen, 2019; Loft & Jensen, 2020). It has been reported that nurses who remain in practice want to create something good for themselves—they want to improve themselves as human beings to become better persons, indicating they are oriented towards themselves (Kristoffersen & Friberg, 2015). Nurses want to have a feeling of being useful and valuable (Söderbacka et al., 2021) and identify with the need to stay up to date professionally, which means it is important for them to use their knowledge and skills. Moreover, nurses want to be taken care of by being recognized for their work (Kristoffersen, 2021). However, nurses become frustrated as they are not able to care for themselves and they push aside their own needs in favour of others (Andrews et al., 2020). This means that nurses have to balance between the good they want for themselves and what they face in daily nursing practice (Kristoffersen, 2019). Researchers call for greater recognition of nurses becoming aware of what they want and need for themselves, as this may have relevance for their remaining in practice (Egan et al., 2019; Mills et al., 2018; Wood, 2018).

Unfortunately, one in five nurses in Norway leaves nursing during the first 10 years of practising their profession (Skjøstad et al., 2017). It is estimated that Norway will have a shortage of about 28,000 nurses by 2035 (Hjelmås et al., 2019), while the international nursing shortage is estimated to be 7.6 million by 2030 (World Health Organization, 2020). However, it is worth noting that Norwegian school nurses tend to remain in practice (Ekornrud & Thonstad, 2021; Lassemo & Melby, 2020). Nevertheless, due to the establishment of new positions and natural retirement, Norway is now seeing a lack of school nurses (Hjelmås et al., 2019). A shortage of school nurses is also widespread in the United States and Europe (Jansen et al., 2018; Willgerodt et al., 2018). In Norway, school nurses are Registered Nurses holding an advanced degree in Public Health Nursing, employed in municipalities that are legally obliged to offer a school health service for both public and private schools, from elementary to high school. The school nurses' responsibility, like their counterparts in other parts of the world, involves promoting health and preventing disease in relation to school children's lives and upbringing and providing both acute and temporary health care for school children (Baltag et al., 2015; Glavin et al., 2014; Jansen et al., 2018). It is notable that nursing students seem to prefer working with children and young people (Øster et al., 2017; Rognstad et al., 2004). Research in the field of school nursing shows that daytime work and an independent professional role are appreciated and seems to be specific reasons for nurses entering school nursing (Foley et al., 2004; Junious et al., 2004; Smith & Firmin, 2009). On an everyday basis, school nurses meet school children face‐to‐face in individual consultations or group activities, in classrooms and through digital media (Sherwin, 2019) and want to be someone who makes a difference in the lives of the children (Jameson & Bowen, 2020; Smith & Firmin, 2009). Nevertheless, it is well documented that school nurses experience high demands, such as heavy workload and complex work situations (Heggestad et al., 2021; Klein & Evans‐Agnew, 2019; Sendall et al., 2014). A nursing practice with high demands prompts school nurses to seek new knowledge and professional growth to improve and fulfil their tasks (Dahl & Clancy, 2015; Glavin et al., 2014; Jönsson et al., 2019; Junious et al., 2004). School nurses who manage their work tasks are more satisfied and likely to remain in practice (Jameson & Bowen, 2020) and research has also demonstrated that school nurses' remaining in practice seem to revolve around taking a stand for what is good for themselves (Mæland et al., 2021).

Although the above literature review indicates what seems to be involved in what school nurses strive to achieve for themselves, it does not fully explore more specifically what this is. As there seems to be a link between the goods nurses strive to achieve on their own behalf and their remaining in practice, it is of interest to study what the school nurses strive to achieve for themselves. This can be made visible and thus possible to grasp in what they orient towards in their school nursing. School nursing is a field of nursing that differs somewhat from other nursing practice. However, it is of interest to study a group of nurses who tend to remain in practice, as their experience related to what they strive to achieve for themselves in order to remain in practice may be unique. Focusing on what school nurses strive to achieve for themselves can also contribute to informing why nurses in other fields of nursing remain in practice.

### 1.1 Aim

The aim of this study was to describe and interpret what it is school nurses strive to achieve for themselves in order to remain in practice.

## 2 THE STUDY

### 2.1 Design

This qualitative study has a hermeneutic approach (Taylor, 1985). The approach was suitable as it involved to describe what the text says and to interpret the text to make the underlying meaning clear. This implied a circular movement between parts and the whole of the data material. The study was designed to comprise two, individual in‐depth interviews with each participant (Seidman, 2013) and a phenomenological hermeneutic method for analysis (Lindseth & Norberg, 2004). This method opened up for an analysis of experiential meaning, related to what it is the school nurses strived to achieve for themselves in order to remain in practice.

### 2.2 Method

#### 2.2.1 Participants

A purposive sample was applied (Polit & Beck, 2018), with the inclusion criteria: advanced education as public health nurse, full or almost full‐time work (75%–100%) in a current position as a school nurse and a minimum of 3 years' work experience as a school nurse. The first author contacted the head of school health services in 39 municipalities in the south of Norway for the distribution of written information about the study. The head of the school health services invited school nurses who met the inclusion criteria to participate in the study. In total, 15 school nurses from 12 municipalities contacted the first author and consented to participate. All the participants were female and aged between 33 and 60 years (median 48). Their work experience ranged from 11 to 39 years as a registered nurse, whereof 4–20 years as a public health nurse in school health services. Eleven of the participants had additional qualifications related to mental health, psychosocial work, paediatrics, midwifery, intensive care, leadership, family therapy or counselling.

#### 2.2.2 Data collection

The data were collected through interviews that began with an introductory open‐ended question (Seidman, 2013), implying the participants were asked to talk freely about what they strive to achieve for themselves in order to remain in practice. Follow‐up questions were posed: “Could I hear more about what you are striving to achieve for yourself?” “What significance does this have for you related to your remaining in practice?” “What does this mean for you?” “What does this imply for you?” and “Is there something more you can say about what it is you are striving to achieve for yourself in order to remain in practice?” The second interview was conducted 2–9 weeks after the first interview and the participants were asked the same introductory open‐ended question. The average length of each interview was 70 min for the first interview and 40 min for the second. The participants chose the time and place for the interviews, which were held in undisturbed locations, mainly in their place of work. All interviews were audiotaped and transcribed verbatim. The data material comprised 30 interviews and were collected between April and August 2018.

#### 2.2.3 Data analysis

In the first analysis phase of the phenomenological hermeneutic method, which involved a naïve reading, the first author listened to the recorded interviews and repeatedly read the interview transcripts with an open mind to gain a preliminary understanding of the participants' descriptions of what it is they strive to achieve for themselves in order to remain in practice. The preliminary understanding guided the subsequent analysis and was formulated as follows:

What the school nurses strived to achieve for themselves was to do their best for the school children. At the same time, they were aware of being kind to themselves to be able to remain in practice.

The second phase involved de‐contextualizing the text and identifying meaning units (Lindseth & Norberg, 2004). The analysis was guided by analytical questions based on the preliminary understanding: “What is involved in doing their best for the school children?” and “What do they talk about when they mention that they were aware of being kind to themselves?” More specific analytical questions were asked to condense the text and identify meaning units, for example, “What are they oriented towards in order for them to remain in practice?” “What do they consider is worth realizing to remain in practice?” Reflective questions were also posed to the text: “What does this involve?” “What is this about?” “What does this mean?” The analysis involved a search for similarities and differences in the text for further condensations. In this way, the preliminary understanding was validated. All authors discussed the meaning reflected in the condensed text to reach a common understanding and thematize the findings. The themes were formulated with the use of active verbs and matched the school nurses' everyday language as closely as possible.

In the last phase, the preliminary understanding and the themes were re‐read and reflected upon in relation to the study's aim and background in order to reveal and formulate an overall interpretation, presented as a comprehensive understanding (Lindseth & Norberg, 2004).

#### 2.2.4 Ethical considerations

The study was approved by the Norwegian Centre for Research Data (project 59195). Oral and written information about the study was given to the heads of school health services and school nurses who consented to participate. They were informed of the estimated time for two interviews, their right to withdraw from the study at any time and ensured confidentiality, as data were managed following research ethical guidelines (World Medical Association, 2013). All participants signed an informed consent before the first interview.

#### 2.2.5 Rigour

Rigour was ensured by following the criteria for trustworthiness: credibility, confirmability, dependability and transferability (Lincoln & Guba, 1985). Credibility was ensured by conducting two in‐depth interviews with each participant, giving them time for their reflections to mature and to deepen and elaborate on what they had said in their first interview. Interviews on two separate occasions contributed to prolonged engagement, which facilitated an open and trustful atmosphere for the participants to talk freely. To ensure confirmability, the first and last authors discussed the transcript of the first interview to consider proximity in the interview situation and whether relevant questions were asked to achieve rich data. No changes were made to the interview guide. Although all the interviews were performed by the first author, all authors reviewed transcribed interviews and took part in all analytical stages by discussing the condensed texts until we reached consensus on the analysis and formulated themes. Dependability is provided through a clear presentation of the study aim, data collection and analysis. The research process has been described and findings are presented with participants' quotes to facilitate external judgement and transferability to other nursing contexts. The project follows the Consolidated Criteria for Reporting Qualitative Research (COREQ; Tong et al., 2007) in design, methodological considerations and presentation of findings.

## 3 FINDINGS

The thematic analysis resulted in the identification of three themes: (1) trusting your own professional ability, (2) aspiring to appreciation and (3) accomplishing self‐care.

### 3.1 Trusting your own professional ability

Trusting their own professional ability involved the school nurses being confident in their professional knowledge and skills. To remain in practice, feeling confident was of value to the nurses, as it enabled them to do their best. For the most part, the nurses were the only health professionals available at the schools, which means that they were alone in making decisions about how their nursing practice should be performed. Trusting themselves as school nurses, they sought professional gravitas. However, sometimes they wondered whether they had adequate knowledge and relevant skills for each situation. One of the nurses said: “I can have strange thoughts like, was this good enough?” (13). Another nurse emphasized the importance of professional gravitas:

It is important for me to be able to stand tall in what I do. Feeling confident, being able to withstand a storm is a good thing. It gives me the courage to speak up, believe in myself and my ability as a nurse.(5)

Trusting her own professional ability encouraged this nurse to speak up and let her professional voice be heard. She wanted to perceive herself as capable of “withstanding a storm,” a metaphor that represented that she stood “steady” through difficulties. Standing steady enabled her to perform as a qualified nurse who acted courageously for a child's rights, for example, in confronting parents with concerns about their home situation. In confronting parents, it was imperative for the nurses to trust their own professional ability to speak up for what was considered needed for the child. One nurse, who described herself as generally conflict‐shy, allowed her shyness to give way to trust that she had what it takes to be a school nurse:

When there's a child I'm concerned about, I fight on the barricade for them, I never give up. I must be as tough as I can, to do what's best for the child. It's a challenge, but I have to face the difficulties, I have to take a phone call with the parents and talk directly with them about something unpleasant.(12)

The quote indicates that trusting her professional ability contributed to strengthening her and conquering her shyness for the child's benefit, which enabled her to realize the purpose of her practice.

### 3.2 Aspiring to appreciation

The school nurses aspired to appreciation, which means they strived to acknowledge themselves as a nurse. The nurses considered this to be of value in regard to their remaining in practice. Acknowledging themselves involved their practising nursing that was relevant and needed by the school children. The school nurses wanted to support and protect vulnerable children by doing what they could for them. It made the nurses recognize themselves as someone who make a positive change and confirmed their significance as nurses. One nurse said: “Making a difference for school children is a chance to do something really crucial, which makes me feel that what I do is significant and important” (8). For this nurse doing “something crucial” made her feel appreciated. For another nurse, acknowledging herself by making a difference for the school children was considered more valuable to her remaining in practice than earning her salary:

For me, it is valuable to know my job is important. That I can make a difference, a positive change for someone means more to me than earning my salary. My point is, I want to do what I can to ensure the kids are well, that they have a good upbringing and feel safe at home. I feel appreciated when the child gives me a hug and I understand that what I do means a lot to them.(4)

Aspiring to appreciation was connected to having a reputation for high‐quality practice. A nurse working at a school in a rural location strived to achieve a good reputation and be known for the way she practised nursing and dealt with people:

As a school nurse in a smaller village I become extremely visible, I'm *the* school nurse. I want to be liked and have a good reputation. However, not at any cost – I want to do a proper job, meet people with respect, generosity and warmth.(9)

This nurse wanted to be recognized for being a warm, caring and generous school nurse in the village, implying she valued being admired for her professional values and accepted for the effort of performing a proper job.

### 3.3 Accomplishing self‐care

The theme “accomplishing self‐care” describes what the school nurses strived to achieve for themselves, considering what was necessary for them to manage their working day and remain in practice. To manage their working days it was essential they prioritized what was good and valued themselves as a nurse. One nurse said:

Naturally I want to help the children, but ‐ it's also about what I need to be able to hold on: I do need a break and peace in my mind and time to reflect. I struggle to find time to sit down for a cup of coffee in the teachers' staff room, which feels like a luxury. Also, it is important for me to meet other school nurses, which is like a space where I can breathe. I know it's my responsibility to make time for these breaks myself, and I have to become better at prioritizing it.(13)

This nurse associated “having a break,” meeting other school nurses or time to reflect as a “luxury.” Doing good for herself was essential for her to “breathe,” and without “breathing,” it was not possible to hold on. Having a break was nonetheless hard to prioritize and thus, a prerequisite was to set limits for herself and consider which tasks were relevant to accomplish. She continued:

I cannot say yes to everything I'm asked to take part in. If I'm asked to teach, I use quite a lot of time to prepare, which means that I have to clear my schedule of other appointments. I always have to juggle and reduce the number of things I say yes to.(13)

To accomplish self‐care, the nurses took control of what they agreed to be engaged in by taking a stand for caring for themselves. This involved the nurses being aware of and verbalizing what they needed for themselves. Taking a stand was required, as they often felt divided between the child and themselves. It was difficult to refuse or close the door in front of those crying or asking for their help. They had to balance self‐care with care for the children when they felt drawn to prioritize the child, at the same time having awareness of the importance of accomplishing self‐care. In such situations, they sometimes contrasted the value of what they needed for themselves with the value of taking care of the child. Having to make time for oneself was reflected in one nurse's unspoken wish that children might sometimes miss their appointments:

If I'm to remain as a school nurse, there must be a balanced workload. I try to avoid filling my days with too many appointments. Deep down I know it will not work being overloaded. I need time to reflect, and I cannot push myself too hard. I get exhausted if I overbook and think “I'll manage.” Actually, I do have too much work, and sometimes I hope one child is away from school, not able to come …(9)

Without accomplishing self‐care, the school nurses could exceed their capacity as a nurse, which one of them described as “a feeling of drowning” (8). Thus, self‐care protected them from exhaustion. Exhaustion could be the outcome if the nurses ignored themselves and thereby risked self‐sacrifice. Accomplishing self‐care prevented a feeling of exhaustion and strengthened the nurses' resolve to make time for what they needed to do for themselves.

## 4 COMPREHENSIVE UNDERSTANDING AND DISCUSSION

The study shows that “trusting their own professional ability,” “aspiring to appreciation” and “accomplishing self‐care” was what the school nurses strive to achieve for themselves. This was reflected in their choices and actions, which involved doing their best for the school children while simultaneously being aware of the need to be kind to themselves. These findings can be comprehensively interpreted in light of the Canadian philosopher Charles Taylor's (1989) description of goods of higher value. Taylor describes goods of higher value to be attributed qualitative “worth or importance” compared with other goods (Taylor, 1989, p. 63). Goods of higher value can be goals, desires or ends that are “worthy or desirable” “to a greater degree” than other goods (Taylor, 1989, p. 20). Taylor states that human beings have the capacity to make distinctions between different goods and consider some of overriding value. He also states that such goods provide “an orienting sense” in life as “the connection between seeing the good and being moved by it cannot be broken” (Taylor, 1989, p. 74). The school nurses in this study articulated what they strived to achieve for themselves, which pointed to what was of worth to them in daily nursing in order to remain in practice. Thus, realizing what was of worth to them can be regarded as a good of higher value.

Realizing what it is the school nurses strived to achieve for themselves in order to remain in practice implies they trust their own professional ability. The school nurses attributed value to being confident in their knowledge and skills and this enabled them to do their best in school nursing by “acting courageously” and in keeping with how they wanted to practise as a nurse. It is no surprise that such a good was of value to the school nurses. Being a school nurse calls for professional confidence based on a broad range of general knowledge and skills, and a sense of being capable affects their ability to accomplish complex work tasks (Heggestad et al., 2021; Jönsson et al., 2019; Klein & Evans‐Agnew, 2019). The school nurses often operate alone and lack daily contact with other health professionals (Glavin et al., 2014). Dahl and Clancy, for instance, characterize school nurses as “specialized generalists” (Dahl & Clancy, 2015, p. 684), which emphasizes that professional gravitas is highly relevant. School nurses who perceive they have a high level of professional ability may experience lower levels of burnout and are more likely to remain in practice (Jameson & Bowen, 2020). This finding is supported by previous research indicating that nurses in other fields want to feel confident, have professional courage (Loft & Jensen, 2020) and keep their professional knowledge and skills up to date (Kristoffersen, 2021).

In addition, realizing what it is the school nurses strived to achieve for themselves in order to remain in practice implies they aspire to appreciation. It involved the school nurses valuing and acknowledging themselves, doing so by practising nursing that was relevant and needed by the school children—they wanted to make a difference to the school children. Making a positive change for school children was a great source of recognition and gratification for the school nurses and is documented as a reason for their remaining in practice (Jameson & Bowen, 2020; Sendall et al., 2014; Smith & Firmin, 2009). Moreover, for them, aspiring for appreciation was more valued than earning their salary in order to remain in practice. This demonstrates that goods other than financial benefits can be seen as reasons for remaining in school nursing, which is a finding in line with previous research (Foley et al., 2004; Junious et al., 2004). Researchers have indicated that nurses in other fields of the nursing experience feeling special by making a difference to others (Lindh et al., 2009; Söderbacka et al., 2021) and experiencing being of worth influences their remaining in practice (Alilu et al., 2016; Kristoffersen & Friberg, 2015; Loft & Jensen, 2020).

Realizing what it is the school nurses strived to achieve for themselves in order to remain in practice implies they accomplish self‐care. This revolved around what they attributed value to and was needed for them to manage their working day. What they valued as good for themselves as a nurse could involve quite small things, such as making time for reflection or a coffee break. As this current study indicates, accomplishing self‐care was necessary as it delimited a risk of exhaustion, a finding supported by previous research in other nursing contexts, which have documented that nurses' awareness of their own needs strengthens their ability to provide nursing care for patients (Egan et al., 2019; Wood, 2018). Previous research has also demonstrated that nurses' self‐care and doing what is good for themselves are related to their health and well‐being and thus their ability to manage practising daily nursing care (Andrews et al., 2020; Mills et al., 2018).

Nevertheless, the current study's comprehensive understanding does not indicate that what the school nurses strived to achieve for themselves were goods that were never at odds with each other. Taylor states that goods might come into conflict with one another, as there can be several goods of “ultimate significance” “which we feel we cannot repudiate but which seem to demand incompatible things of us” (Taylor, 1985, p. 236). Taylor (1985, 1989) points to the importance of being sensitive about what goods are worthy or desirable to a greater degree than other goods in the relevant situation. He states that this involves qualitative discrimination in prioritizing and taking a stand for realizing what is of higher value in each case. The school nurses sometimes had to prioritize which good to realize. In particular, realizing the accomplishment of self‐care could involve omitting aspiring to appreciation implying acknowledging themselves as a nurse and their reputation for high‐quality practice. Sometimes the school nurses had to say “no” to school children who asked for their help. This demonstrates that the school nurses qualitatively discriminated between goods to consider which was of higher value to realize in each situation in order to remain in practice. It has been stated that to remain in practice, nurses have to balance the contrasts in nursing practice—the goods of higher value that they strive to achieve and the challenges they face (Kristoffersen, 2019). The current study adds to this finding by demonstrating more specifically what goods the nurses within the field of school nursing strived to achieve for themselves. It also demonstrates that the challenge they faced might have involved qualitative discrimination between what good is of higher value in each situation. This required a balancing act. Nevertheless, what the school nurses attributed value to themselves gave an orienting sense and formed their lives as nurses: They remained in school nursing—a finding in line with Kristoffersen and Friberg (2018), who documented that nurses' remaining in other fields of nursing revolves around realizing what is of qualitative worth in their life as a nurse. Moreover, this study indicates what school nurses identify as good for themselves, which may relate to realizing themselves in practice (Mæland et al., 2021). What it is nurses strive to achieve for themselves should therefore not be underestimated but acknowledged as a good of higher value to realize for remaining in practice.

### 4.1 Strengths and limitations

This study's qualitative design implied a description of school nurses' subjective experience that has been comprehensively interpreted from the data. We acknowledge that one limitation might concern the inclusion criterion limiting participation to experienced school nurses. As a result, the average age of the participants was 48. It is conceivable that younger nurses, who tend to have a high degree of turnover, could have reported different aspects of what they strive to achieve for themselves to remain in practice than those described by the participants. However, the population of younger nurses holding an advanced degree in public health nursing in Norway is limited. Likewise, the lack of male participants can be explained by the very small population of male school nurses in Norway. Additionally, as school nursing differs somewhat from other fields of nursing, another study context or sample might have resulted in other findings. Nevertheless, this study's findings may be transferrable to nurses working in other contexts, as the comprehensive understanding is an overall interpretation that can provide insight about nurses who tend to remain in practice and what they strive to achieve for themselves. Due to the discrepancies in the timespan between interviews one and two, some participants experienced less time between the two interviews, which may have affected the maturation of their reflections. The data material nevertheless consisted of two individual interviews with each participant, which we consider generated rich and nuanced data material with high information power relevant to the study's aim (Malterud et al., 2015).

## 5 CONCLUSION

This study has identified that what school nurses strive to achieve for themselves is “trusting their own professional ability,” “aspiring to appreciation” and “accomplishing self‐care.” Realizing what they strive to achieve for themselves is comprehensively understood as a good of higher value relevant for their remaining in practice. To remain in practice, the school nurses seem to maintain a balance by deciding which good was of the highest value to realize in each situation.

A nursing practice that encourages and supports nurses in realizing trusting their own professional ability, aspiring to appreciation and accomplishing self‐care to manage their working day can influence nurses' remaining in practice. This can, for example, involve providing nurses with relevant knowledge and training, acknowledging them for their work and effort and helping them become aware of and prioritize what is good and what they attribute value to themselves. However, more research is needed to study what nurses in other contexts or demographics strive to achieve for themselves in nursing practice and how to support nurses in prioritizing time and ability to realize this, which in turn might have relevance for their remaining in practice.

## ETHICAL APPROVAL

The study was approved by the Norwegian Center for Research Data (project 59195). The study conforms to research ethical guidelines in the Declaration of Helsinki. Participants gave their informed consent and were informed about their right to decline, as well as their anonymity was guaranteed.

## Data Availability

The data that support the findings of this study are available from the corresponding author upon reasonable request.
